# Nanotechnology in cervical cancer immunotherapy: Therapeutic vaccines and adoptive cell therapy

**DOI:** 10.3389/fphar.2022.1065793

**Published:** 2022-12-16

**Authors:** Xuyan Zhou, Haiying Lian, Hongpeng Li, Meiling Fan, Wei Xu, Ye Jin

**Affiliations:** ^1^ School of Pharmacy, Changchun University of Chinese Medicine, Changchun, China; ^2^ Gynecology Department, Affiliated Hospital of Changchun University of Chinese Medicine, Changchun, China

**Keywords:** cervical cancer, nanotechnology, immunotherapy, therapeutic vaccine, adoptive cell therapy, nanocarriers

## Abstract

Immunotherapy is an emerging method for the treatment of cervical cancer and is more effective than surgery and radiotherapy, especially for recurrent cervical cancer. However, immunotherapy is limited by adverse effects in clinical practice. In recent years, nanotechnology has been widely used for tumor diagnosis, drug delivery, and targeted therapy. In the setting of cervical cancer, nanotechnology can be used to actively or passively target immunotherapeutic agents to tumor sites, thereby enhancing local drug delivery, reducing drug adverse effects, achieving immunomodulation, improving the tumor immune microenvironment, and optimizing treatment efficacy. In this review, we highlight the current status of therapeutic vaccines and adoptive cell therapy in cervical cancer immunotherapy, as well as the application of lipid carriers, polymeric nanoparticles, inorganic nanoparticles, and exosomes in this context.

## Introduction

Cervical cancer is caused by the growth of abnormal cells spreading to other parts of the body. It is one of the most common female reproductive malignancies (Bedell et al., 2020). Globally in 2020, there were 604,000 new cases of cervical cancer and 342,000 deaths. Cervical cancer is the fourth-leading cause of cancer incidence and mortality among women worldwide after breast cancer, lung cancer, and colon cancer (World Health Organization, 2022). Cervical cancer has a high incidence and tends to be younger. Therefore, cervical cancer has become a substantial public health concern (Ibrahim Khalil et al., 2022).

Smoking, poor hygiene, early initiation of sexual intercourse, and having multiple sexual partners are risk factors for cervical cancer (Small et al., 2017). The main cause of cervical cancer is persistent high-risk human papillomavirus (HPV) infection. E6 and E7 are HPV oncoproteins that interact with the tumor suppressors p53 and retinoblastoma protein (pRb), respectively. Activation of apoptotic pathways is disrupted by these interactions, which subsequently promote cell proliferation and ultimately increase progression of HPV-associated malignancies (Jee et al., 2021). At present, more than 200 HPV subtypes have been identified that primarily infect cells in mucous membranes and the epidermis (Perri et al., 2003). These subtypes can be classified into high-risk and low-risk types depending on whether or not they promote the development of malignant lesions. Low-risk HPV types do not cause cancer but can cause genital warts. In contrast, only 12 high-risk HPV types are oncogenic (Dell et al., 2008; Aranda-Rivera et al., 2021). The most prevalent high-risk HPV type is HPV 16, which is associated with approximately 50% of cervical cancer cases, followed by HPV 18 and HPV 31 (Stuebs et al., 2021).

Depending on the diagnosis, clinicopathological features, and other risk factors of the disease stage, surgery or a combination of chemotherapy and radiotherapy is included as first-line therapy treatment (Cohen et al., 2019). Due to the expansion of early detection methods and enhanced efficacy of surgery and radiotherapy, the prognoses of patients with early cervical cancer has been significantly improved. However, most conventional treatments can only achieve therapeutic effects on local solid tumors. The survival rates of patients with advanced, recurrent, or metastatic cervical cancer are still poor (Lee et al., 2019; Liontos et al., 2019). Indeed, the 5-year survival rate of patients with cervical cancer is 60%–70% in countries with high human development index (HDI). In comparison, the survival rate drops to less than 20% in countries with low HDI (Wakeham and Kavanagh, 2014). Most antineoplastic drugs have severe side effects, limiting their maximum tolerated dose. Together with the development of drug resistance, this factor leads to decreased therapeutic efficacy (Ghalkhani et al., 2022). Therefore, it is crucial that more effective treatments should be developed. Immunotherapy can eliminate tumors and prevent tumor recurrence by eliciting long-term effects on immune memory (Chen et al., 2022).

However, in the setting of cervical cancer, immunotherapy is still associated with clinical challenges such as low immunogenicity, inefficient targeting, and immunotoxicity (Song et al., 2015, 20). With the rapid development of nanotechnology, clinical diagnoses and treatments for cervical cancer have greatly improved. For example, nanotechnology-mediated delivery of drugs can increase drug solubility, control drug release rates *in vivo*, and improve drug stability. Also, nanotechnology-mediated delivery systems have the ability to deliver one or more treatments (e.g., chemotherapeutic drugs and/or immunotherapeutic agents) to the lesion site. Additionally, combining nanotechnology with imaging modalities can facilitate visualization of the drug delivery process (Irvine and Dane, 2020; Martin et al., 2020). Furthermore, mesoporous silica and gold nanoparticles can enhance the sensitivity of clinical cervical cancer diagnoses and enable early detection and timely treatment (Palantavida et al., 2013; Yin et al., 2020). In this review, we discuss the utility of therapeutic vaccines and adoptive cell therapy in cervical cancer immunotherapy, as well as summarize the application of nanotechnology in this context.

## Immunotherapy and cervical cancer

**FIGURE 1:**
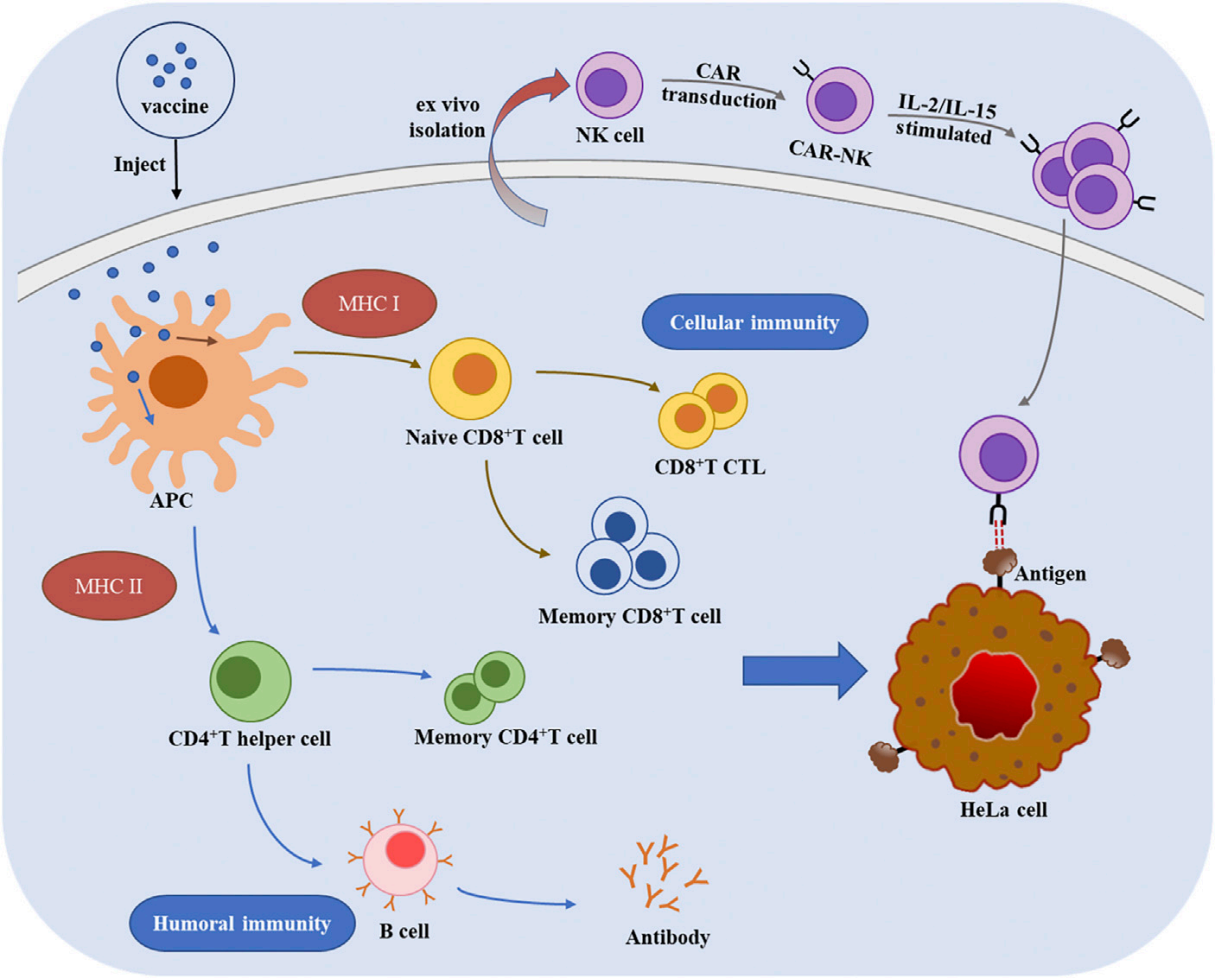
The mechanisms of therapeutic vaccines and adoptive cell therapy for cervical cancer.

Immunotherapy leverages immunological principles and methods to activate and enhance the body’s immune system. Immunotherapy can enhance the ability of immune system to recognize, attack, and neutralize tumor cells, thereby inhibiting tumor growth (Duan et al., 2019; Zhang et al., 2022). The mechanisms underlying therapeutic vaccines and adoptive cell therapy are shown in Figure 1.

The ideal antigens for cervical cancer therapeutic vaccines are the E6 and E7 viral oncoproteins, which are constitutively expressed by HPV-infected host cells (Miles et al., 2017). Following exposure to co-stimulatory molecules, antigen-presenting cells (APCs) can uptake pathogens and present pathogenic peptides on their surfaces, which can then be recognized by the major histocompatibility complex (MHC) (Purcell et al., 2007; Stark et al., 2019). Live-vector vaccines, such as the bacterial vectors *Listeria monocytogenes* (Lm), *Lactococcus lactis*, *Lactobacillus plantarum*, and *Lactobacillus casei* are highly immunogenic (Bermúdez-Humarán et al., 2004; Cortes-Perez et al., 2005; Sewell et al., 2008). ADXS11-001 is a live attenuated Lm vaccine that generates an immune response against the HPV 16 E7 oncoprotein. Preliminary results from phase III clinical trials have demonstrated the efficacy of this vaccine against recurrent or persistent cervical cancer (Vonsky et al., 2019). The currently available viral vaccines that target E6 and E7 antigens include adenovirus, alphavirus, Venezuelan equine encephalitis virus (VEER), and cowpox virus, among others (Liu et al., 2000; Cassetti et al., 2004; Hsieh, 2004; Gomez-Gutierrez et al., 2007). For example, Cassetti et al. (Cassetti et al., 2004) assembled HPV 16 E6/E7 genes into a VEER vector *via* point mutations in order to treat an HPV 16-related mouse tumor model. This study showed that a cytotoxic T lymphocyte (CTL) response against E7 was induced, resulting in tumor regression.

Peptide/protein-based vaccines have the following characteristics: easy production, favorable safety profiles, and storage stability (Vonsky et al., 2019). Phase II clinical trials of the SGN-00101 vaccine (composed of HPV 16 E7 and *Mycobacterium bovis* heat shock protein) have shown that it can induce the regression of grade II and III cervical intraepithelial neoplasia (Roman et al., 2007). Unlike peptide-based vaccines, protein-based vaccines contain all antigenic epitopes of E6 and E7 and are not restricted by MHC class I. However, both vaccines exhibit low immunogenicity and poor stability *in vivo*. Lipids or other adjuvants should be added to enhance their immune efficacy (Menderes et al., 2016; Smalley Rumfield et al., 2020).

Nucleic acid-based vaccines can be classified as DNA or RNA vaccines. The vaccines inhibit carcinogenesis by maintaining immunogenicity (e.g., by mutating or recombining oncogenes) (Choi et al., 2020; Turinetto et al., 2022). However, the vaccines containing intact E6/E7 gene fragments carry the risk of cell transformation. Specific human leukocyte antigen epitopes can be expressed by mutating the p53/pRB binding site in E6/E7 or by changing the gene sequence, but does not produce antigen protein to circumvent this hazard (Öhlschläger et al., 2006; Brinkman et al., 2007). Nucleic acid vaccines are not only stable and easily produced, but also can be administered repeatedly. However, they are less immunogenic and diffuse, and show low immunogenicity. Adjuvants, combination treatments, and multiple vaccination methods should be needed to enhance immunogenicity (Menderes et al., 2016).

Unlike the other three vaccines, dendritic cell (DC)-based vaccines are the only vaccines that can activate naive T cells. *In vitro*, DCs can be sensitized with viral peptides, DNA, or RNA. Subsequently, HPV antigens are loaded in a vaccine and inject into the body to increase the efficacy of antigen presentation (Kumbhari et al., 2020; Fu et al., 2022). DC-based vaccines can also be used as delivery adjuvants to enhance T cell-mediated immunity in HPV-associated lesions. However, due to their complexity and costly production, these vaccines cannot be produced on large scale (Zeng et al., 2018).

Adoptive cell therapy (ACT) involves the amplification of highly effective tumor-reactive cells collected from patients through *in vitro* activation or genetic engineering transformation. ACT is a form of personalized medicine that can amplify the immunity of many cell types, including chimeric antigen receptor-natural killer cells (CAR-NKs), cytokine-induced killer cells (CIKs), tumor-infiltrating lymphocytes (TILs), T cell receptor-T cells (TCR-Ts), and chimeric antigen receptor-T cells (CAR-Ts) (Ye et al., 2017; Yeh et al., 2017; Rohaan et al., 2019). The preparation of T cell receptors are complicated *in vitro*, which are required presentation by the MHC system. So it is difficult to produce on a large scale (Dai et al., 2019). In contrast, CAR-NKs can kill a broad spectrum of tumor cell types efficiently and without pre-sensitization. Studies have shown that increasing proportion of NKs in the external environment of cervical cells can ablate HPV colonization and suppress infection in tissues, thereby preventing cervical intraepithelial neoplasia from progressing (Lucena et al., 2016).

## Application of nanotechnology in immunotherapy of cervical cancer

**FIGURE 2:**
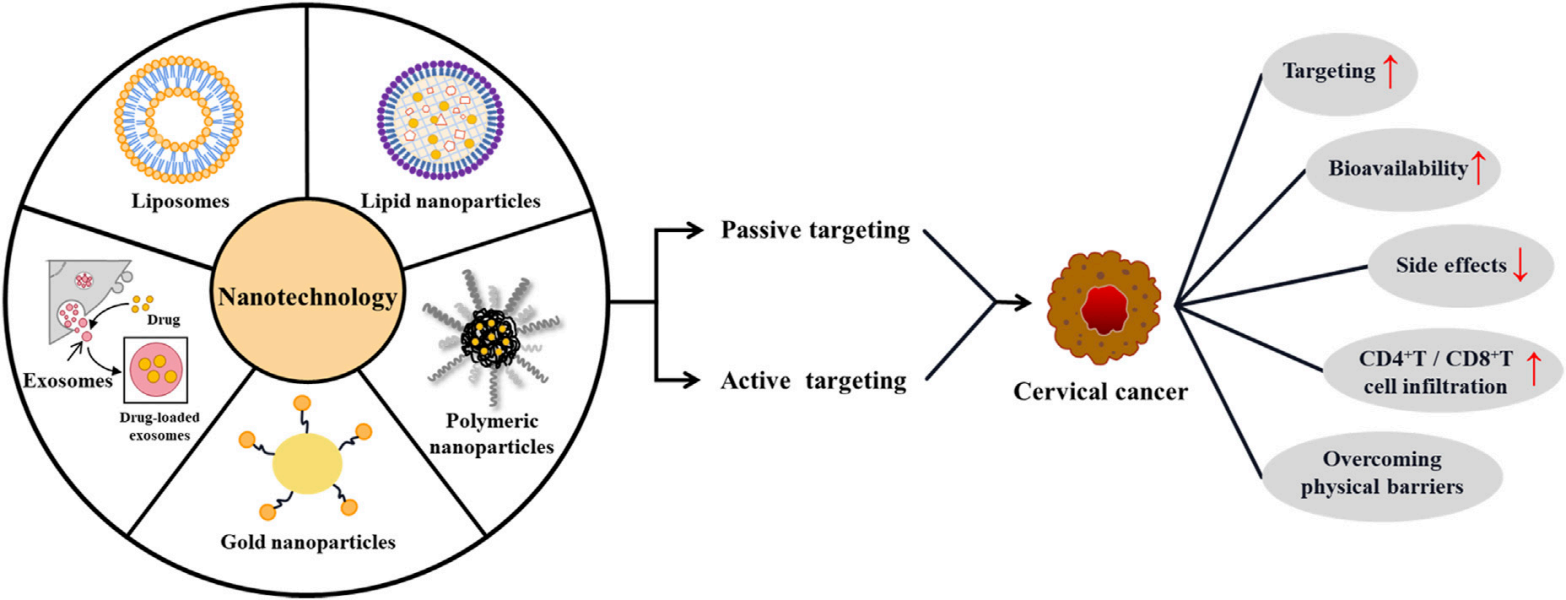
Nanotechnology-mediated immunotherapy for cervical cancer.

Nanotechnology can be used for passive targeting systems because of the enhanced permeability and retention (EPR) effect. In addition, it can enhance the ability of active targeting *via* surface modification of targeted molecules. Applying nanotechnology enables drugs, antibodies, and immunomodulators to be enriched at the tumor site (Shi and Lammers, 2019; Liu et al., 2021). Subsequently, it reverses immunosuppression in the tumor microenvironment (TME) and activates tumor-specific cytotoxic T cells, thereby improving the efficacy of immunotherapy (Figure 2) (Zhang et al., 2012; Batty et al., 2019). Currently, nanotechnology carriers commonly used for cervical cancer immunotherapy include lipid carriers, polymeric nanoparticles, inorganic nanoparticles, and exosomes. The advantages and limitations of these methods are shown in Table 1.

**TABLE 1:** Advantages and limitations of nanotechnology.

Nanotechnology		Advantages	Limitations	References
Lipid-based nanocarriers	PEGylated liposome	Improve bioavailability	Low cellular uptake	(Ishida et al., 2003; Mishra et al., 2004)
		Biodegradability	Accelerated blood clearance phenomenon	
		Biocompatibility		
	Tween 80 modified liposomes	High physical stability	Easy to hemolysis phenomenon	(Thumrongsiri et al., 2022)
		Enhanced blood-brain barrier permeability	Only suitable for intramuscular injection	
	Lipid Nanoparticles	High encapsulation efficiency	Difficult to achieve industrial production	(Yang et al., 2013)
		Improve bioavailability		
		High physical stability		
		Biocompatibility		
Polymeric nanoparticles	PLGA nanoparticles	Biocompatibility	Low drug loading	(Pandita et al., 2015)
		Enhance controlled and prolonged effects of drug release		
	PAMAM nanoparticles	High transfection efficiency	Existence of cytotoxicity	(Choi et al., 2004; Li et al., 2021)
		Proton sponge effect		
Inorganic nanomaterials	AuNPs	High physical stability	Toxic effect on the biological system	(Artiga et al., 2019)
		Ultra-small size, large surface area-to-volume ratio and high reactivity	Low permeability	
		Easy surface modification		
	Mesoporous silica nanoparticles	Biocompatibility	Uncertainty between particle size and toxicity	(Shahabi et al., 2015)
		Large surface area-to-volume ratio		
		Porous structure		
Exosomes	Genetic engineering modified exosomes	Biocompatibility	The drug loading process was not controllable	(Stremersch et al., 2016)
		High biological permeability	Existence of cytotoxicity	
		High targeting		
	Chemically modified exosomes	Simple preparation	Functional proteins that may damage membrane surface	(Haney et al., 2015)
		Controllable process		

### Lipid-based nanocarriers

Lipid-based nanocarriers are safe and exhibit good biocompatibility. Liposomes are closed, spherical vesicles with a bilayer membrane structure, which are consisted of natural phospholipids (soy phospholipids and lecithin), synthetic phospholipids, or cholesterol (Luiza Ribeiro de Souza et al., 2012). Liposomes can be prepared *via* the film dispersion, reverse evaporation, chemical gradient, hot-melt, and solvent injection methods. Depending on the preparation technique, single unilamellar vesicles (SUVs), large unilamellar vesicles (LUVs), or multivesicular liposomes (MLVs) can be obtained. The particle size of liposomes is typically ranging 20 nm to a few microns (Al-Jamal et al., 2008; Filipczak et al., 2020). Because of the amphipathic nature of liposomes, water-soluble and lipid-soluble components (e.g., chemotherapeutic drugs, plant extracts, and immune cytokines) can be encapsulated in liposomes. Meanwhile, specific ligands of tumor cell receptors are loaded onto the liposome surfaces in order to improve targeting ability (Filipczak et al., 2020). For example, Chen et al. (Chen and Huang, 2008) developed an improved liposome N-[1-(2,3-Dioleoyloxy) propyl]-N,N,N-trimethylammonium chloride (DOTAP)/E7 lipopeptide vaccine to treat HPV-positive tumors. Compared to natural E7, this vaccine reduced the amount of antigen required to inhibit tumor growth and improved the production of functional CTL responses. Additionally, Karimi et al. (Karimi et al., 2020) combined an late HPV capsid protein (L1)/E6/E7 recombinant gene with Archaeosomes to produce an Archaeosome-L1/E6/E7 vaccine for *in vivo* evaluation. They found that Archaeosomes resulted in an approximately 3-fold increase in apoptosis levels compared to L1/E6/E7 recombinant gene. It also promoted immune responses to DNA vaccines and exhibited inhibitory activity on tumor cells.

Lipid nanoparticles contain natural or synthetic solid lipids, liquid lipids, and surfactant carrier molecules. Drugs are adsorbed or encapsulated in the lipid core to form particles of 50–1,000 nm in size, which in turn can be prepared by extrusion, ultrasonic, or homogenization methods (Sheoran et al., 2022). Due to the use of electroporation during the transfer of CAR-encoding mRNA into T cells, the integrity CAR-T plasma membrane were compromised, which reduces the next gene expression (Guevara et al., 2020). Patel et al. (2022) developed ionizable lipid nanoparticles to deliver CAR-encoding mRNA into T cells, producing functional CAR-T cells with enhanced tumoricidal activity. The core cavity is more suited to the encapsulation of oligonucleotide drugs, whereas other lipid carriers are more suitable for small molecule inhibitors or lipids. For example, Kranz et al. (2016) found that using lipid carriers such as 1,2-dioleoyl-sn-glycero-3-phosphoethanolamine, [1-(2,3-dioleyloxy) propyl]-N,N,N-trimethylammonium chloride, and cholesterol. The uptake and expression of different macrophage populations in lymphoid organs can be enhanced by modulating the negative net charge of the nanoparticles.

### Polymeric nanoparticles

Polymeric nanoparticles are artificial spherical nanoparticles that generally range from 10 to 500 nm in size. They can be prepared by numerous methods including emulsification, aggregation, coacervation, and the supercritical antisolvent technique, among others (Ali, 2020). Polymeric nanoparticles have different classifications according to their structural and functional properties, such as number of polymeric monomers can be classified as unimolecular and multimolecular. Block polymers can be classified as single- and composite-component polymers. Also, most polymeric materials have large molecular weights (Castro et al., 2022). During the formation of nanoparticles, hydrophobic drugs can be encapsulated in the inner core thereby protecting them in the systemic circulation (Kim et al., 2016). Moreover, the hydrophilic shells of polymeric nanoparticles can be modified with active targeting ligands, which can aggregate drugs at tumor sites and improve the therapeutic potentials of chemotherapeutic drugs (Ke et al., 2014). Polymeric materials are characterized by good biodegradability and biocompatibility. Such as polylactic acid, poly (D,L-lactide-co-glycolic acid) (PLGA), and polyglutamic acid which have been extensively investigated for cancer prevention and immunotherapy (Pan et al., 2013; Sadr et al., 2018). For example, co-delivery of an HPV 16 E7 DNA vaccine with interleukin-12 (IL-12) using chitosan increased DNA vaccine E7-specific lymphocyte proliferation and CTL activity (Tahamtan et al., 2018). Moreover, the combined action of chitosan and IL-2 increased HPV 16 L1 antibody titers and mucosal protection (Ma et al., 2015). Additionally, Nagapoosanam et al. (Nagapoosanam et al., 2019) loaded targeted hTERT and siRNA onto PLGA nanoparticles. The nanocarriers enabled the stable release of siRNA for 72 h and significantly accelerated HeLa cell apoptosis. Finally, Galliverti et al. (Galliverti et al., 2018) combined an HPV E7 synthetic long peptide with ultrasmall polymeric nanoparticles to promote the infiltration of CD8^+^ T cells. Accompanied aggregation of regulatory T cells was not observed, contributing to the enhanced antineoplastic effects of this vaccine.

### Inorganic nanoparticles

Inorganic nanomaterials are composed of inorganic substances with structural units of at least one nanoscale dimension in three-dimensional space (typically 1–100 nm) (Meena et al., 2020). Inorganic nanoparticles can be prepared *via* the chemical co-precipitation, microemulsion, and pyrolysis methods (Islam et al., 2012). Inorganic nanoparticles are widely used in drug delivery and tumor therapy due to their unique physical and chemical properties, ease of surface modification, and good biocompatibility (Hao et al., 2020). The primary inorganic nanoparticles used in the context of HPV-related diseases are copper oxide, silica, gold, and zinc oxide (Bayda et al., 2018). For example, Yang et al. (2021) developed a novel nanocomposite, polyethyleneimine-modified dendritic mesoporous silica nanoparticle that was loaded with microRNA-125a. This particle showed excellent cellular uptake capacity in the TC-1 cervical cell line. Intratumoral injection synergistically enhanced immune responses and reversed the immunosuppression. Also, it enhanced the infiltration of NKs and CD8^+^ T cell. In addition, Yi et al. (2016) developed suppression-targeted gold nanoparticles (AuNPs) by loading monodispersed unimer polyion complexes—which consisted of therapeutic siRNA, cyclic Arg-Gly-Asp, and blocking cations—onto AuNPs. These particles not only effectively delivered the HPV E6-targeting siRNAs, but also suppressed xenograft tumors derived from HeLa cells. Finally, Dey et al. (2020) found that chitosan-conjugated copper oxide nanoparticles could inhibit the proliferation of cervical cancer cells and promote the infiltration of CD4^+^ T cells by triggering humoral IgG-dependent immune responses and activating immune cells to induce cellular immunity.

### Exosomes

Exosomes are small intranuclear particles with a lipid bilayer structure. These extracellular vesicles typically exhibit a cup-shaped morphology and a diameter of approximately 30–150 nm (Raposo and Stoorvogel, 2013). Exosomes can be secreted by various cell types such as tumor cells, lymphocytes, and DCs. They are ubiquitous in body fluids such as blood, urine, and cerebrospinal fluid (Rashed et al., 2017; Wu et al., 2021). Exosomes can facilitate signal transduction between immune cells, thereby activating downstream effector cells. It enables the presentation of tumor-specific antigens to the immune system and inhibits tumor immune escape (Rodrigues et al., 2018). Bioactive components (e.g., drugs, nucleic acids, and proteins) can be encapsulated into exosomes *via* membrane fusion, electroporation, genetic engineering, and ultrasound loading to achieve targeted drug delivery to specific cells or tissues (Chakravarti et al., 2020). For example, Chen et al. (2018) developed a DC-derived exosome (Dexo) loaded with the E7 (49–57) peptide. Dexo effectively induced the cytolytic activity of CD8^+^ T cells against TC-1 cervical cancer cells *in vitro*. Also, it induced the proliferation of CD8^+^ T cell and the secretion of interferon γ. In addition, the Dexo vaccine promoted E7 antigen-induced immune responses in the splenocytes of immunized mice. Furthermore, Cenik et al. (2022) demonstrated that docetaxel-loaded exosomes (Exo-Doc) could reduce docetaxel dosage and toxicity. At the same time, they induced mitochondrial apoptosis in HeLa cells and increased the metastasis of resistant cells. Finally, Roy et al. (2022) identified adipocyte-derived stem cell exosomes (ACS-exos). Delivery of microRNA-7 to cervical cancer cells with ACS-exos induced downregulation of X-linked inhibitor of apoptosis protein. This study showed that the successful isolation and transfection of exosomes are critical to the use of exosomes for cancer therapy. Moreover, exosome-loaded nucleic acids can be delivered to target cells, leading to altered protein expression.

## Conclusion

In this review, we discuss the application of therapeutic vaccines and adoptive cell therapy to cervical cancer. However, immunotherapy for cervical cancer is marred by challenges such as low immunogenicity, poor targeting, and immunotoxicity. In our summary, we found that the combination of nanotechnology and immunotherapy can eliminate the adverse effects of immunotherapy agents and improve their therapeutic efficacy in cervical cancer. However, these nanotherapeutic agents are still in the preclinical stages of development (Table 2). Due to uncertainty concerning the EPR effect in tumor tissues of different patients, it is unknown whether nanotechnology will improve drug delivery in tumor tissues. Moreover, many challenges remain with respect to translating industrial products to the clinic. Therefore, this review describes a foundation for nanotechnology-mediated cervical cancer immunotherapy. Further research pertaining to the large-scale production, safety, and stability of nanocarrier-loaded immunotherapeutics is needed.

**TABLE 2:** Clinical application of nanotechnology-based therapy for cervical cancer.

Therapeutic agents	Therapeutic method	Pathways	Phase/Status	Trial number	References
Liposomal HPV-16 E6/E7 Multipeptide Vaccine PDS0101, Cisplatin	Immunotherapy and chemoradiation	JAK/STAT	II/Recruitment	NCT04580771	(Gutiérrez-Hoya and Soto-Cruz, 2020; Smalley Rumfield et al., 2020)
Pegylated liposomal doxorubicin hydrochloride, Carboplatin	Chemotherapy	Bcl-2	I-II/Completed	NCT00032162	(du Bois et al., 2007; Xia et al., 2020)
Nanoparticle Albumin-Bound Rapamycin	Immunotherapy	PI3K/Akt/mTOR	Early I/Completed	NCT02646319	(Jain et al., 2021; Zhang et al., 2021)
BIND-014 (docetaxel nanoparticles for injectable suspension)	Chemotherapy	PI3K	II/Terminated	NCT02479178	(Von Hoff et al., 2016; Liu et al., 2019)
Irinotecan liposome, apatinib, PD-1 antibody	Chemoradiation, immunotherapy	PI3K/Akt/mTOR, PD-1/PD-L1	II/Completed	NCT04569916	(Boussen et al., 2010; Guo et al., 2020; He et al., 2021)
